# Obesity Accelerates Age Defects in Mouse and Human B Cells

**DOI:** 10.3389/fimmu.2020.02060

**Published:** 2020-09-02

**Authors:** Daniela Frasca, Bonnie B. Blomberg

**Affiliations:** ^1^Department of Microbiology and Immunology, University of Miami Miller School of Medicine, Miami, FL, United States; ^2^Sylvester Comprehensive Cancer Center, University of Miami Miller School of Medicine, Miami, FL, United States

**Keywords:** aging, obesity, B cells, inflammation, antibody responses

## Abstract

Obesity, similar to aging, is associated with chronic low-grade systemic inflammation, known as inflammaging, and represents a significantly higher risk for developing chronic diseases typical of old age. Immune cells are recruited to the obese adipose tissue (AT) by chemotactic molecules secreted by non-immune and immune cells in the AT, both contributing to the release of several pro-inflammatory mediators that fuel local and systemic inflammation, to the refractory response of immune cells to further *in vivo* and *in vitro* stimulation and to the induction of autoimmune B cells with potentially pathogenic repertoires. In terms of molecular mechanisms involved, leptin, an adipokine secreted primarily by adipocytes, has been proposed to be involved in the reduced generation of protective antibodies, and in the increased generation of autoimmune antibodies, further supporting the concept that obesity accelerates age defects. Leptin has also been shown to induce intrinsic B cell inflammation and B cell immunosenescence. The results presented in this review highlight the importance of weight reduction programs to improve immunity and reduce the risk for developing chronic diseases in obese and older individuals.

## Introduction

Obesity, defined as body-mass index (BMI) ≥ 30 kg/m^2^ by CDC and WHO, is an increasing health concern that affects young (1) and older adults (2), and has reached pandemic proportions. Individuals with obesity are at higher risk for developing chronic diseases typical of old age such as cardiovascular disease (3), Type-2 Diabetes Mellitus (T2DM) (4–6), cancer (7), psoriasis (8), atherosclerosis (9), inflammatory bowel disease (10). Obesity-induced metabolic changes cause tissue dysfunction, disruption of the integrity of lymphoid tissues, and decreased leukocyte development and function, all leading to reduced protective immunity. One of the reasons is because obesity, similar to aging, is an inflammatory condition associated with chronic low-grade systemic inflammation, inflammaging (11), which is negatively associated with a functional immune system, healthspan and longevity in both mice and humans (12). All immune cells contribute to the inflammatory status of obese individuals, and increased frequencies of pro-inflammatory macrophages (13, 14), T cells (15, 16), and B cells (17, 18) have been reported. Decreased frequencies of regulatory B cells have also been shown in the blood of individuals with obesity (19).

## The Obese Adipose Tissue (At)

Obesity is characterized by increased mass of the AT. The AT is a storage of nutrients and an active endocrine and immunological tissue. The AT is composed of adipocytes and a mixture of mesenchymal, endothelial and immune cells, known as the Stromal Vascular Fraction (SVF) (17, 20). Under conditions of over-nutrition, the AT changes from a condition of insulin sensitivity (IS) to a condition of insulin resistance (IR) that is occurring in parallel with the expansion of adipocyte mass, remodeling of extracellular matrix components (collagens, elastins and the associated blood vasculature) and increased secretion of pro-inflammatory mediators (cytokines, chemokines, adipokines, leukotrienes), involved in the recruitment of immune cells to the AT. Chronic inflammation in the AT contributes to inflammaging and leads to increased IR in obesity (21). IR also increases with age (22, 23), and is associated with high serum levels of glucose and free fatty acids (FFAs), and markers of metabolic inflammation, metaflammation (24), that fuels inflammaging and promotes aging, diseases and death.

Altered innate and adaptive immune responses occur in the AT under conditions of over-nutrition (17, 20). Mouse (25, 26) and human (27, 28) results have shown that immune cells are recruited to the obese AT by chemotactic molecules secreted by non-immune and immune cells in the AT, generating a positive feedback loop in which a large number of pro-inflammatory mediators are secreted, contributing to local and systemic inflammation. The obese AT of mice (25, 26) and humans (28, 29) also secretes antibodies that have been shown to be pathogenic (25) in mouse studies. These antibodies are IgG2c, a subclass associated with murine autoimmunity (26). These antibodies are specific for AT-derived “self” proteins and nucleic acids, including dsDNA, found increased in the plasma of elderly individuals, suggesting that obesity may drive the secretion of autoimmune antibodies during aging. This may occur even in elderly lean individuals, due to the deposition of fat on internal organs which is known to increase during aging. An age-associated increase in ectopic deposit of triglycerides in several tissues (liver, muscle, heart, pancreas, kidney) (30–34) and blood vessels (35) has indeed been reported and the word “TOFI” (thin-outside-fat-inside) has been coined to identify lean individuals with abnormal abdominal adiposity and inflammaging. Moreover, age-associated changes in abundance, distribution and cellular composition of the AT have been reported and shown to accelerate the onset of age-associated diseases (36, 37). Computational tomography scans have shown that with age subcutaneous AT (SAT) mass decreases, whereas visceral AT (VAT) mass increases (38). SAT and VAT are biologically distinct in secretion of pro-inflammatory mediators, with VAT being more inflammatory. Furthermore, secretion of adipokines by cells in the AT is regulated by nutrients, and these responses are increased with aging (39).

Senescent cells accumulate in the AT of aging mice and humans (40). Senescent cells are characterized by the irreversible arrest of cell proliferation due to different types of stress, and by the secretion of factors that constitute the senescence-associated secretory phenotype (SASP), consisting of soluble pro-inflammatory molecules, soluble receptors, growth factors and extracellular matrix macromolecules (41). The age-dependent accumulation of senescent cells is a favorable environment for the development of inflammatory-based age-associated diseases and for this reason several strategies have been developed to decrease accumulation of senescent cells in tissues and suppress the SASP with the aim to delay the onset of age-associated diseases (42, 43).

## Effects of Obesity on Mouse B Cells

Obesity, similar to aging, impairs several aspects of B cell biology. In mice fed a high-fat diet (HFD), early B cell development is characterized by decreased frequencies of B cell subsets in the bone marrow (BM) and reduced expression of early lymphoid commitment markers such as the B cell transcription factor PAX5 (44). Mechanistic experiments using co-cultures of BM cells with the OP9 stromal cell line have shown that BM adipocytes secrete soluble factors that drive the development of myeloid-derived suppressor cells (MDSCs) (45, 46). MDSC inhibition of B lymphopoiesis is mediated by MDSC-derived IL-1β and the inflammatory molecule complex called calprotectin, suggesting that these may be therapeutic targets for the restoration of B lymphoiesis in obesity and aging.

Splenic B cell function is also affected by HFD. Initial studies have indicated that mice fed HFD secrete more pro-inflammatory cytokines (IL-6/TNF-α) than B cells from mice fed normal-fat diet (NFD), thus contributing to the higher levels of systemic inflammation observed in mice fed HFD (47) and in aged mice (48). B cells from HFD mice, in turn, induce changes in the AT and promote adipocyte hyperthropy, hyperglycemia and IR and induce T cell and macrophage inflammation (25). Mice lacking B cells (μMT mice) (49) have reduced IR and glucose intolerance.

Splenic B cells from obese mice have been shown to be pathogenic, as demonstrated by adoptive transfer experiments in which B cells from HFD mice, transferred into B^null^ mice, induce IR and glucose intolerance only if recipients are on HFD, suggesting that the development and/or maintenance of pathogenic B cells requires exposure to HFD (25). B cells from HFD mice influence the function of T cells and macrophages and induce secretion of IFN-γ and TNF-α, respectively, two crucial cytokines involved in the establishment of IR. IgG antibodies isolated from the serum of HFD, but not NFD, mice are mediators of IR and glucose intolerance and induce FcγR-mediated activation of macrophages and consequent TNF-α secretion. B cell depletion using anti-CD20 antibodies decreases obesity-induced glucose abnormalities and ameliorates metabolic disease. All these results were among the first to show the fundamental role of B cells in the pathogenesis of obesity-associated IR.

While the spontaneous secretion of pathogenic IgG antibodies increases in the spleen of HFD mice (25), as well as in the spleen of aged mice (26), the secretion of protective IgG antibodies decreases (44, 50). It has been shown that even mice fed a Western Diet (that provides a moderate but lower quantity of fat than the HFD), showed significantly lower influenza-specific titers as compared to NFD mice after infection with the influenza virus A/Puerto Rico/8/34 (44). In the same study, it was shown that mice fed HFD together with DHA (docosahexaenoic acid), an essential FA with immunostimulatory function, whose serum levels are low in obesity, had improved influenza-specific antibody responses, suggesting that DHA may be used as a therapeutic strategy to increase humoral immunity.

Also, mucosal B cells from HFD mice regulate obesity-induced IR (51). IgA secreting B cells, as well as secreted IgA antibodies, are significantly reduced in the colon of HFD vs. NFD mice, similar to what has been observed in the colon of aged mice (52). IgA deficiency, specifically in intestinal B cells, deteriorates glucose homeostasis in HFD but not NFD mice, further confirming that the negative regulation of glucose metabolism needs exposure to HFD. IgA antibodies control host-microbiome homeostasis and provide a barrier for microbial and/or ingested antigens that may translocate from the gut into the blood, inducing inflammatory responses. IgA antibodies also regulate lipid absorption from the gut.

The characterization of potentially pathogenic B cell repertoires, performed using high-throughput Ig sequencing from several tissues of mice fed HFD and NFD, has shown that HFD significantly changes the biochemical properties of Ig heavy-chain complementarity-determining region-3 (CDRH3) sequences, with IgA antibodies being characterized by shorter and highly hydrophobic CDRH3 (53). HFD is also associated with higher frequencies of unmutated IgA. These changes occur in B cells from the gut and the AT, suggesting the possibility of a gastrointestinal-AT immune axis shaped by HFD. Surprisingly, similar gene rearrangements were found in B cells from the gut, AT and peritoneal cavity of several individual mice, suggesting that affinity maturation may have occurred in these tissues in a similar antigen-specific way.

B cells infiltrate the AT under obesity conditions (25, 26, 54), recruited by several chemotactic signals including those generated by the interaction of the leukotriene B4 with its receptor. Inhibition of this interaction has been shown to reduce B cell recruitment and activation and to mitigate the contribution of B cells to local inflammation and IR (55). AT-associated B cells are highly inflammatory and secrete several pro-inflammatory mediators (cytokines, chemokines, adipokines). It has recently been shown that aging further increases the expansion of these AT resident B cells, through the activation of the NLRP3 inflammasome, a major regulator of inflammaging and age-associated metabolic disorders, likely due to AT-associated metabolic and mitochondria dysfunction and increased production of mitochondrial reactive oxygen species (54). Our studies on mice fed HFD have confirmed the above findings and have shown that the increased size of the AT, increased infiltration of immune cells and increased secretion of pro-inflammatory mediators induce a powerful feed-forward loop of inflammation, both locally and systemically, that are responsible for the refractory response of immune cells to further *in vivo* and *in vitro* stimulation. In particular, we have shown that the AT directly impairs B cell function by changing the composition of the B cell pool and inducing higher frequencies of pro-inflammatory B cells (26), and similar results have been observed in old mice (56).

## Effects of Obesity on Human B Cells

Studies on B cell development in the human BM have shown that soluble factors secreted by the adipocytes inhibit early stages of B lymphopoiesis, with the inhibition occurring at the common lymphoid progenitor to pre/pro-B cell stage (57), suggesting that the age-related decline in B lymphopoiesis is due at least in part to an increase in BM adipocytes, and an increase in adipocyte-derived factors (IL-1β) that directly inhibit B lymphopoiesis.

Obesity decreases B cell function in humans as well, and it is associated with impaired B cell responses to infections and vaccines (58–60). Our results in humans have demonstrated that obesity-associated defects in class switch recombination (CSR) and somatic hypermutation (SHM), two processes necessary for the generation of class switched high affinity secondary antibodies (61), are due to reduced expression of activation-induced cytidine deaminase (AID), the enzyme of CSR and SHM, and E47, encoded by the E2A gene, a key transcription factor regulating AID (62). Both AID and E47 are decreased in B cells isolated from the blood of obese young and elderly individuals as compared to lean controls. Importantly, the response of elderly lean individuals was not different from that of young obese individuals, supporting the hypothesis that obesity accelerates age defects in B cells. At least one mechanisms involved in the decrease of AID/E47 in B cells from obese vs. lean individuals was the decreased expression of phosphorylated-AMPK (59), up-stream of phosphorylated-p38 MAPK, crucial for E47 activation, as previously shown in murine B cells (63). Another mechanism was associated with the increased expression of the inflammatory micro-RNA (miR)-155 and miR-16 in unstimulated B cells from obese vs. lean individuals, with miR-155 binding the 3'-untranslated region (3'-UTR) of AID mRNA and miR-16 binding the 3'-UTR of E47 mRNA, inducing their degradation (59). These results recapitulate what we have initially shown in our studies on the effects of aging on B cell function in which both AID and E47 were found decreased in mitogen-stimulated B cells from elderly as compared to young individuals (64).

Leptin has been proposed to be at least one molecular mechanism involved in dysfunctional B cell function in individuals with obesity. Leptin is an adipokine secreted primarily by the adipocytes (65) with endocrine and immune functions, whose serum concentration correlates with the amount of body fat and BMI (66). Leptin increases the secretion of pro-inflammatory cytokines by immune cells, and *ob/ob* mice that are leptin-deficient have reduced secretion of Th1 cytokines and increased secretion of Th2 cytokines (67).

**Figure 1:**
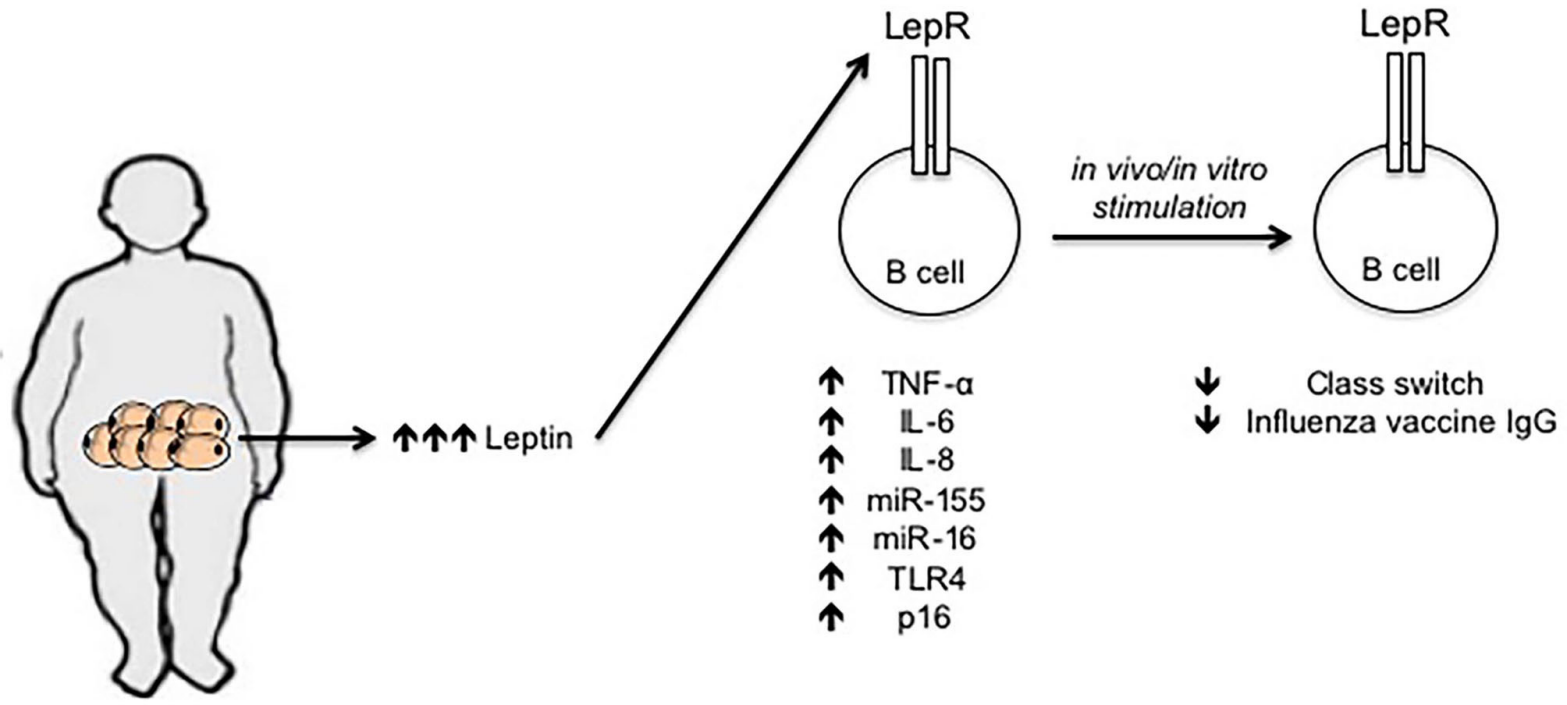
Effects of leptin on human B cell function. Leptin is secreted by adipocytes in the AT. Leptin concentration increases locally and systemically under obesity conditions. Leptin interacts with its receptor on the surface of B cells and increases mRNA expression of several markers of inflammation and cellular senescence, such as TNF-α, IL-6, IL-8, miR-155, miR-16, TLR4, and p16. The expression of these mediators in unstimulated B cells is negatively associated with the response of the same B cells after *in vivo*/*in vitro* stimulation, e.g., with the influenza vaccine.

Leptin levels in the serum of young obese individuals are comparable to those in the serum of elderly lean individuals (68), and we have recently demonstrated that incubation of B cells from young lean individuals with leptin decreases class switch and influenza vaccine-specific IgG antibodies, similar to the levels observed in B cells from young obese and from elderly lean individuals, further supporting the concept that obesity accelerates age defects. Leptin also increases the frequencies of pro-inflammatory B cells and induces intrinsic B cell inflammation, measured by mRNA expression of several pro-inflammatory markers associated with immunosenescence, the expression of which before stimulation negatively correlates with the response of the same B cells after stimulation (68). Previously published data have also shown that leptin activates human peripheral blood B cells to secrete the pro-inflammatory cytokines TNF-α and IL-6 (69, 70). Figure 1 summarizes our recently published results on the effects of leptin on B cell function.

Obesity increases blood frequencies of the subset of B cells called late memory, tissue-like or double negative (DN) B cells (CD19+CD27-IgD-), that represents the most inflammatory B cell subset, also increased in the blood of elderly individuals (71, 72) and of autoimmune patients (73–75). DN B cells do not proliferate and do no make antibodies to “new” antigens, but they secrete antibodies specific for autoantigens known to increase with age and autoimmune diseases, such as the “self” antigens dsDNA and Malondealdehyde, a product of lipid peroxidation and a marker of oxidative stress (76). DN B cells that secrete anti- “self” antibodies are characterized by the membrane phenotype CD95+CD21-CD11c+, and by the spontaneous expression of the transcription factor T-bet (29, 72), two features of human B cells present not only in patients with autoimmune diseases but also in individuals with chronic inflammatory conditions, including aging and obesity (77, 78).

Previously published results have indicated that the plasma of obese individuals with IR contains autoantibodies specific for intracellular proteins, ubiquitously expressed in tissues including pancreas, nervous tissues, muscle, AT, as well as in immune cells (25), suggesting the release of “self” antigens under obesity conditions in insulin target tissues. More recently, we have shown that the human obese AT contributes to increased secretion of adipocyte-specific IgG antibodies and this occurs without any stimulation, likely because the ongoing process of cell death in the obese AT leads to the release of “self” antigens, that are almost exclusively intracellular or cell-associated, able to chronically stimulate B cells (28). Adipocyte-specific IgGs secreted in the obese AT are significantly correlated with those present in the plasma (79).

DN B cells are the cells secreting anti- “self” antibodies in the human obese AT. DN B cells are significantly increased in frequencies in the SVF of the human obese AT. Autoimmune antibody secretion occurs after a metabolic adaption that allows DN B cells to activate oxidative phosphorylation, aerobic glycolysis and fatty acid oxidation, as well as pathways that mitigate stress and cell death, leading to a better survival and function in the hostile pro-inflammatory environment of the obese AT (29). Under these conditions, metabolic reprogramming represents a significant advantage, allowing cells to adapt and survive even when they encounter metabolically restrictive conditions, such as hypoxia, nutrient deprivation and exposure to inflammatory stimuli, as it happens during obesity and aging.

## Conclusions

The mechanisms for the down-regulation of mouse and human B cell responses by obesity and aging are in large part overlapping. Obesity accelerates inflammaging and induces metabolic, physiological, and functional changes in immune cells that lead to defective humoral immunity. The results in this review highlight the importance to prevent obesity as a way to improve immunity and reduce the risk for developing chronic diseases typical of old age.

## Author Contributions

DF wrote the mini review. DF and BB were involved in funding acquisition, reviewing, and editing the manuscript.

## Conflict of Interest

The authors declare that the research was conducted in the absence of any commercial or financial relationships that could be construed as a potential conflict of interest.

## References

[1] Hruby A Hu FB. The epidemiology of obesity: a big picture. Pharmacoeconomics. (2015) 33:673–89. 10.1007/s40273-014-0243-x 25471927 PMC4859313

[2] Collaborators GBDO Afshin A Forouzanfar MH Reitsma MB Sur P Estep K. Health effects of overweight and obesity in 195 countries over 25 years. N Engl J Med. (2017) 377:13–27. 10.1056/NEJMoa1614362 28604169 PMC5477817

[3] Apovian CM Gokce N. Obesity and cardiovascular disease. Circulation. (2012) 125:1178–82. 10.1161/CIRCULATIONAHA.111.022541 22392865 PMC3693443

[4] Hotamisligil GS. Inflammation and metabolic disorders. Nature. (2006) 444:860–7. 10.1038/nature05485 17167474

[5] Shoelson SE Lee J Goldfine AB. Inflammation and insulin resistance. J Clin Invest. (2006) 116:1793–801. 10.1172/JCI29069 16823477 PMC1483173

[6] Johnson AM Olefsky JM. The origins and drivers of insulin resistance. Cell. (2013) 152:673–84. 10.1016/j.cell.2013.01.041 23415219

[7] Renehan AG Tyson M Egger M Heller RF Zwahlen M. Body-mass index and incidence of cancer: a systematic review and meta-analysis of prospective observational studies. Lancet. (2008) 371:569–78. 10.1016/S0140-6736(08)60269-X 18280327

[8] Setty AR Curhan G Choi HK. Obesity, waist circumference, weight change, and the risk of psoriasis in women: nurses' health study II. Arch Intern Med. (2007) 167:1670–5. 10.1001/archinte.167.15.1670 17698691

[9] Casas R Sacanella E Estruch R. The immune protective effect of the Mediterranean diet against chronic low-grade inflammatory diseases. Endocr Metab Immune Disord Drug Targets. (2014) 14:245–54. 10.2174/1871530314666140922153350 25244229 PMC4443792

[10] Hass DJ Brensinger CM Lewis JD Lichtenstein GR. The impact of increased body mass index on the clinical course of Crohn's disease. Clin Gastroenterol Hepatol. (2006) 4:482–8. 10.1016/j.cgh.2005.12.015 16616354

[11] Franceschi C Bonafe M Valensin S Olivieri F De Luca M Ottaviani E. Inflamm-aging. An evolutionary perspective on immunosenescence. Ann N Y Acad Sci. (2000) 908:244–54. 10.1111/j.1749-6632.2000.tb06651.x 10911963

[12] Ahima RS. Connecting obesity, aging and diabetes. Nat Med. (2009) 15:996–7. 10.1038/nm0909-996 19734871

[13] Kraakman MJ Murphy AJ Jandeleit-Dahm K Kammoun HL. Macrophage polarization in obesity and type 2 diabetes: weighing down our understanding of macrophage function? Front Immunol. (2014) 5:470. 10.3389/fimmu.2014.00470 25309549 PMC4176397

[14] Castoldi A Naffah De Souza C Camara NO Moraes-Vieira PM. The macrophage switch in obesity development. Front Immunol. (2015) 6:637. 10.3389/fimmu.2015.00637 26779183 PMC4700258

[15] Gerriets VA Maciver NJ. Role of T cells in malnutrition and obesity. Front Immunol. (2014) 5:379. 10.3389/fimmu.2014.00379 25157251 PMC4127479

[16] Aguilar EG Murphy WJ. Obesity induced T cell dysfunction and implications for cancer immunotherapy. Curr Opin Immunol. (2018) 51:181–6. 10.1016/j.coi.2018.03.012 29655021 PMC6338436

[17] Frasca D Blomberg BB Paganelli R Aging, Obesity, and Inflammatory Age-Related Diseases. Front Immunol. (2017) 8:1745 10.3389/fimmu.2017.01745 29270179 PMC5725402

[18] Frasca D Diaz A Romero M Blomberg BB. Ageing and obesity similarly impair antibody responses. Clin Exp Immunol. (2017) 187:64–70. 10.1111/cei.12824 27314456 PMC5167022

[19] Garcia-Hernandez MH Rodriguez-Varela E Garcia-Jacobo RE Hernandez-De La Torre M Uresti-Rivera EE Gonzalez-Amaro R. Frequency of regulatory B cells in adipose tissue and peripheral blood from individuals with overweight, obesity and normal-weight. Obes Res Clin Pract. (2018) 12:513–9. 10.1016/j.orcp.2018.07.001 30115554

[20] Grant RW Dixit VD. Adipose tissue as an immunological organ. Obesity. (2015) 23:512–8. 10.1002/oby.21003 25612251 PMC4340740

[21] Yuan M Konstantopoulos N Lee J Hansen L Li ZW Karin M Reversal of obesity- and diet-induced insulin resistance with salicylates or targeted disruption of Ikkbeta. Science. (2001) 293:1673–7. 10.1126/science.1061620 11533494

[22] Sepe A Tchkonia T Thomou T Zamboni M Kirkland JL. Aging and regional differences in fat cell progenitors - a mini-review. Gerontology. (2011) 57:66–75. 10.1159/000279755 20110661 PMC3031153

[23] Barzilai N Ferrucci L Insulin resistance and aging: a cause or a protective response? J Gerontol A Biol Sci Med Sci. (2012) 67:1329–31. 10.1093/gerona/gls145 22859390

[24] Hotamisligil GS. Inflammation, metaflammation and immunometabolic disorders. Nature. (2017) 542:177–85. 10.1038/nature21363 28179656

[25] Winer DA Winer S Shen L Wadia PP Yantha J Paltser G. B cells promote insulin resistance through modulation of T cells and production of pathogenic IgG antibodies. Nat Med. (2011) 17:610–7. 10.1038/nm.2353 21499269 PMC3270885

[26] Frasca D Diaz A Romero M Vazquez T Blomberg BB. Obesity induces pro-inflammatory B cells and impairs B cell function in old mice. Mech Ageing Dev. (2017) 162:91–9. 10.1016/j.mad.2017.01.004 28111127 PMC5560850

[27] Zamboni M Rossi AP Fantin F Zamboni G Chirumbolo S Zoico E. Adipose tissue, diet and aging. Mech Ageing Dev. (2014) 136–7:129–37. 10.1016/j.mad.2013.11.008 24321378

[28] Frasca D Diaz A Romero M Thaller S Blomberg BB. Secretion of autoimmune antibodies in the human subcutaneous adipose tissue. PLoS ONE. (2018) 13:e0197472. 10.1371/journal.pone.0197472 29768501 PMC5955545

[29] Frasca D Diaz A Romero M Thaller S Blomberg BB. Metabolic requirements of human pro-inflammatory B cells in aging and obesity. PLoS ONE. (2019) 14:e0219545. 10.1371/journal.pone.0219545 31287846 PMC6615614

[30] Ryan AS Nicklas BJ. Age-related changes in fat deposition in mid-thigh muscle in women: relationships with metabolic cardiovascular disease risk factors. Int J Obes Relat Metab Disord. (1999) 23:126–32. 10.1038/sj.ijo.0800777 10078845

[31] Machann J Thamer C Schnoedt B Stefan N Stumvoll M Haring HU. Age and gender related effects on adipose tissue compartments of subjects with increased risk for type 2 diabetes: a whole body MRI/MRS study. MAGMA. (2005) 18:128–37. 10.1007/s10334-005-0104-x 16001284

[32] Saisho Y Butler AE Meier JJ Monchamp T Allen-Auerbach M Rizza RA. Pancreas volumes in humans from birth to age one hundred taking into account sex, obesity, and presence of type-2 diabetes. Clin Anat. (2007) 20:933–42. 10.1002/ca.20543 17879305 PMC2680737

[33] Silaghi A Piercecchi-Marti MD Grino M Leonetti G Alessi MC Clement K. Epicardial adipose tissue extent: relationship with age, body fat distribution, and coronaropathy. Obesity. (2008) 16:2424–30. 10.1038/oby.2008.379 18719675

[34] Foster MC Hwang SJ Porter SA Massaro JM Hoffmann U Fox CS. Fatty kidney, hypertension, and chronic kidney disease: the framingham heart study. Hypertension. (2011) 58:784–90. 10.1161/HYPERTENSIONAHA.111.175315 21931075 PMC3204377

[35] Robert L. Aging of the vascular-wall and atherosclerosis. Exp Gerontol. (1999) 34:491–501. 10.1016/S0531-5565(99)00030-3 10817805

[36] Guo SS Zeller C Chumlea WC Siervogel RM. Aging, body composition, and lifestyle: the fels longitudinal study. Am J Clin Nutr. (1999) 70:405–11. 10.1093/ajcn/70.3.405 10479203

[37] Lutz W Sanderson W Scherbov S. The coming acceleration of global population ageing. Nature. (2008) 451:716–9. 10.1038/nature06516 18204438

[38] Folsom AR Kaye SA Sellers TA Hong CP Cerhan JR Potter JD. Body fat distribution and 5-year risk of death in older women. JAMA. (1993) 269:483–7. 10.1001/jama.269.4.483 8419667

[39] Einstein FH Fishman S Bauman J Thompson RF Huffman DM Atzmon G. Enhanced activation of a “nutrient-sensing” pathway with age contributes to insulin resistance. FASEB J. (2008) 22:3450–7. 10.1096/fj.08-109041 18566293 PMC2537426

[40] Ovadya Y Landsberger T Leins H Vadai E Gal H Biran A. Impaired immune surveillance accelerates accumulation of senescent cells and aging. Nat Commun. (2018) 9:5435. 10.1038/s41467-018-07825-3 30575733 PMC6303397

[41] Campisi J. Cellular senescence: putting the paradoxes in perspective. Curr Opin Genet Dev. (2011) 21:107–12. 10.1016/j.gde.2010.10.005 21093253 PMC3073609

[42] Zhu Y Tchkonia T Pirtskhalava T Gower AC Ding H Giorgadze N. The achilles' heel of senescent cells: from transcriptome to senolytic drugs. Aging Cell. (2015) 14:644–58. 10.1111/acel.12344 25754370 PMC4531078

[43] Palacio L Goyer ML Maggiorani D Espinosa A Villeneuve N Bourbonnais S. Restored immune cell functions upon clearance of senescence in the irradiated splenic environment. Aging Cell. (2019) 18:e12971. 10.1111/acel.12971 31148373 PMC6612633

[44] Kosaraju R Guesdon W Crouch MJ Teague HL Sullivan EM Karlsson EA. B cell activity is impaired in human and mouse obesity and is responsive to an essential fatty acid upon murine influenza infection. J Immunol. (2017) 198:4738–52. 10.4049/jimmunol.1601031 28500069 PMC5482422

[45] Kennedy DE Knight KL. Inhibition of B lymphopoiesis by adipocytes and IL-1-producing myeloid-derived suppressor cells. J Immunol. (2015) 195:2666–74. 10.4049/jimmunol.1500957 26268654 PMC4561202

[46] Kennedy DE Knight KL. Inflammatory changes in bone marrow microenvironment associated with declining B lymphopoiesis. J Immunol. (2017) 198:3471–9. 10.4049/jimmunol.1601643 28320833 PMC5435233

[47] Defuria J Belkina AC Jagannathan-Bogdan M Snyder-Cappione J Carr JD Nersesova YR B cells promote inflammation in obesity and type 2 diabetes through regulation of T-cell function and an inflammatory cytokine profile. Proc Natl Acad Sci USA. (2013) 110:5133–8. 10.1073/pnas.1215840110 23479618 PMC3612635

[48] Wu D Meydani SN. Age-associated changes in immune and inflammatory responses: impact of vitamin E intervention. J Leukoc Biol. (2008) 84:900–14. 10.1189/jlb.0108023 18596135 PMC2538592

[49] Kitamura D Roes J Kuhn R Rajewsky K. A B cell-deficient mouse by targeted disruption of the membrane exon of the immunoglobulin mu chain gene. Nature. (1991) 350:423–6. 10.1038/350423a0 1901381

[50] Frasca D Diaz A Romero M Vazquez T Strbo N Romero L. Impaired B cell function in mice lacking perforin-2. Front Immunol. (2020) 11:328. 10.3389/fimmu.2020.00328 32180773 PMC7057857

[51] Luck H Khan S Kim JH Copeland JK Revelo XS Tsai S. Gut-associated IgA(+) immune cells regulate obesity-related insulin resistance. Nat Commun. (2019) 10:3650. 10.1038/s41467-019-11370-y 31409776 PMC6692361

[52] Hagiwara Y Mcghee JR Fujihashi K Kobayashi R Yoshino N Kataoka K. Protective mucosal immunity in aging is associated with functional CD4+ T cells in nasopharyngeal-associated lymphoreticular tissue. J Immunol. (2003) 170:1754–62. 10.4049/jimmunol.170.4.1754 12574339

[53] Pham TD Chng MHY Roskin KM Jackson KJL Nguyen KD Glanville J. High-fat diet induces systemic B-cell repertoire changes associated with insulin resistance. Mucosal Immunol. (2017) 10:1468–79. 10.1038/mi.2017.25 28422186

[54] Camell CD Gunther P Lee A Goldberg EL Spadaro O Youm YH. Aging induces an Nlrp3 inflammasome-dependent expansion of adipose B cells that impairs metabolic homeostasis. Cell Metab. (2019) 30:1024–39 e1026. 10.1016/j.cmet.2019.10.006 31735593 PMC6944439

[55] Ying W Wollam J Ofrecio JM Bandyopadhyay G El Ouarrat D Lee YS. Adipose tissue B2 cells promote insulin resistance through leukotriene LTB4/LTB4R1 signaling. J Clin Invest. (2017) 127:1019–30. 10.1172/JCI90350 28192375 PMC5330737

[56] Frasca D Romero M Diaz A Alter-Wolf S Ratliff M Landin AM. A molecular mechanism for TNF-alpha-mediated downregulation of B cell responses. J Immunol. (2012) 188:279–86. 10.4049/jimmunol.1003964 22116831 PMC3700394

[57] Bilwani FA Knight KL. Adipocyte-derived soluble factor (s) inhibits early stages of B lymphopoiesis. J Immunol. (2012) 189:4379–86. 10.4049/jimmunol.1201176 23002443 PMC3483032

[58] Ovsyannikova IG White SJ Larrabee BR Grill DE Jacobson RM Poland GA. Leptin and leptin-related gene polymorphisms, obesity, and influenza A/H1N1 vaccine-induced immune responses in older individuals. Vaccine. (2014) 32:881–7. 10.1016/j.vaccine.2013.12.009 24360890 PMC3922536

[59] Frasca D Ferracci F Diaz A Romero M Lechner S Blomberg BB. Obesity decreases B cell responses in young and elderly individuals. Obesity. (2016) 24:615–25. 10.1002/oby.21383 26857091 PMC4769695

[60] Zhai X Qian G Wang Y Chen X Lu J Zhang Y. Elevated B cell activation is associated with type 2 diabetes development in obese subjects. Cell Physiol Biochem. (2016) 38:1257–66. 10.1159/000443073 26982979

[61] Muramatsu M Kinoshita K Fagarasan S Yamada S Shinkai Y Honjo T Class switch recombination and hypermutation require activation-induced cytidine deaminase (AID), a potential RNA editing enzyme. Cell. (2000) 102:553–63. 10.1016/S0092-8674(00)00078-7 11007474

[62] Sayegh CE Quong MW Agata Y Murre C. E-proteins directly regulate expression of activation-induced deaminase in mature B cells. Nat Immunol. (2003) 4:586–93. 10.1038/ni923 12717431

[63] Frasca D Romero M Landin AM Diaz A Riley RL Blomberg BB. Protein phosphatase 2A (PP2A) is increased in old murine B cells and mediates p38 MAPK/tristetraprolin dephosphorylation and E47 mRNA instability. Mech Ageing Dev. (2010) 131:306–14. 10.1016/j.mad.2010.02.002 20219523 PMC3223388

[64] Frasca D Landin AM Lechner SC Ryan JG Schwartz R Riley RL. Aging down-regulates the transcription factor E2A, activation-induced cytidine deaminase, and Ig class switch in human B cells. J Immunol. (2008) 180:5283–90. 10.4049/jimmunol.180.8.5283 18390709

[65] Zhang Y Proenca R Maffei M Barone M Leopold L Friedman JM. Positional cloning of the mouse obese gene and its human homologue. Nature. (1994) 372:425–32. 10.1038/372425a0 7984236

[66] La Cava A Matarese G. The weight of leptin in immunity. Nat Rev Immunol. (2004) 4:371–9. 10.1038/nri1350 15122202

[67] Loffreda S Yang SQ Lin HZ Karp CL Brengman ML Wang DJ. Leptin regulates proinflammatory immune responses. FASEB J. (1998) 12:57–65. 10.1096/fasebj.12.1.57 9438411

[68] Frasca D Diaz A Romero M Blomberg BB. Leptin induces immunosenescence in human B cells. Cell Immunol. (2020) 348:103994. 10.1016/j.cellimm.2019.103994 31831137 PMC7002206

[69] Agrawal S Gollapudi S Su H Gupta S. Leptin activates human B cells to secrete TNF-alpha, IL-6, and IL-10 via JAK2/STAT3 and p38MAPK/ERK1/2 signaling pathway. J Clin Immunol. (2011) 31:472–8. 10.1007/s10875-010-9507-1 21243519 PMC3132280

[70] Gupta S Agrawal S Gollapudi S. Increased activation and cytokine secretion in B cells stimulated with leptin in aged humans. Immun Ageing. (2013) 10:3. 10.1186/1742-4933-10-3 23343052 PMC3557206

[71] Frasca D Diaz A Romero M Blomberg BB. Human peripheral late/exhausted memory B cells express a senescent-associated secretory phenotype and preferentially utilize metabolic signaling pathways. Exp Gerontol. (2017) 87:113–20. 10.1016/j.exger.2016.12.001 27931848

[72] Frasca D Diaz A Romero M D'eramo F Blomberg BB. Aging effects on T-bet expression in human B cell subsets. Cell Immunol. (2017) 321:68–73. 10.1016/j.cellimm.2017.04.007 28457482

[73] Wehr C Eibel H Masilamani M Illges H Schlesier M Peter HH. A new CD21low B cell population in the peripheral blood of patients with SLE. Clin Immunol. (2004) 113:161–71. 10.1016/j.clim.2004.05.010 15451473

[74] Adlowitz DG Barnard J Biear JN Cistrone C Owen T Wang W. Expansion of activated peripheral blood memory B cells in rheumatoid arthritis, impact of B cell depletion therapy, and biomarkers of response. PLoS ONE. (2015) 10:e0128269. 10.1371/journal.pone.0128269 26047509 PMC4457888

[75] Claes N Fraussen J Vanheusden M Hellings N Stinissen P Van Wijmeersch B. Age-associated B cells with proinflammatory characteristics are expanded in a proportion of multiple sclerosis patients. J Immunol. (2016) 197:4576–83. 10.4049/jimmunol.1502448 27837111

[76] Niedernhofer LJ Daniels JS Rouzer CA Greene RE Marnett LJ. Malondialdehyde, a product of lipid peroxidation, is mutagenic in human cells. J Biol Chem. (2003) 278:31426–33. 10.1074/jbc.M212549200 12775726

[77] Phalke S Marrack P. Age (autoimmunity) associated B cells (ABCs) and their relatives. Curr Opin Immunol. (2018) 55:75–80. 10.1016/j.coi.2018.09.007 30388513

[78] Cancro MP. Age-Associated B Cells. Annu Rev Immunol. (2020) 38:315–40. 10.1146/annurev-immunol-092419-031130 31986068

[79] Frasca D Diaz A Romero M Garcia D Jayram D Thaller S. Identification and characterization of adipose tissue-derived human antibodies with “anti-self” specificity. Front Immunol. (2020) 11:392. 10.3389/fimmu.2020.00392 32184790 PMC7058997

